# CAG Repeats Determine Brain Atrophy in Spinocerebellar Ataxia 17: A VBM Study

**DOI:** 10.1371/journal.pone.0015125

**Published:** 2011-01-19

**Authors:** Kathrin Reetz, Alexandra Kleiman, Christine Klein, Rebekka Lencer, Christine Zuehlke, Kathrin Brockmann, Arndt Rolfs, Ferdinand Binkofski

**Affiliations:** 1 Department of Neurology, RWTH Aachen University, Aachen, Germany; 2 Institute of Neuroscience and Medicine, Research Center Jülich GmbH, Jülich, Germany; 3 Jülich-Aachen Research Alliance (JARA) Translational Brain Medicine, Aachen, Germany; 4 Department of Neurology, University of Luebeck, Luebeck, Germany; 5 Schilling Section of Clinical and Molecular Neurogenetics, University of Luebeck, Luebeck, Germany; 6 Department of Psychiatry and Psychotherapy, University of Luebeck, Luebeck, Germany; 7 Institute of Human Genetics, University of Luebeck, Luebeck, Germany; 8 Medical Faculty, Albrecht-Kossel Institute for Neuroregeneration, University of Rostock, Rostock, Germany; 9 Division for Cognitive Neurology, RWTH Aachen University, Aachen, Germany; University of Texas MD Anderson Cancer Center, United States of America

## Abstract

**Background:**

Abnormal repeat length has been associated with an earlier age of onset and more severe disease progression in the rare neurodegenerative disorder spinocerebellar ataxia 17 (SCA17).

**Methodology/Principal Findings:**

To determine whether specific structural brain degeneration and rate of disease progression in SCA17 might be associated with the CAG repeat size, observer-independent voxel-based morphometry was applied to high-resolution magnetic resonance images of 16 patients with SCA17 and 16 age-matched healthy controls. The main finding contrasting SCA17 patients with healthy controls demonstrated atrophy in the cerebellum bilaterally. Multiple regression analyses with available genetic data and also post-hoc correlations revealed an inverse relationship again with cerebellar atrophy. Moreover, we found an inverse relationship between the CAG repeat length and rate of disease progression.

**Conclusions:**

Our results highlight the fundamental role of the cerebellum in this neurodegenerative disease and support the genotype-phenotype relationship in SCA17 patients. Genetic factors may determine individual susceptibility to neurodegeneration and rate of disease progression.

## Introduction

The autosomal dominant spinocerebellar ataxias (SCA) are clinically and genetically heterogeneous neurodegenerative disorders. Twenty-eight genetic subtypes have been yet identified, of which seven are caused by expansion of a CAG trinucleotid repeat that encodes a polyglutamine tract in respective proteins [1]. A longer CAG expansion may lead to an earlier onset [2] and a more severe progression of clinical symptoms.

The rare neurodegenerative disorder SCA17 with mainly adult age of onset is clinically highly variable manifesting with diverse features including ataxia and dementia, extrapyramidal movement and neuropsychiatric disorders, as well as seizures. Given the extraordinarily broad clinical spectrum, SCA17 may mimic other neurodegenerative disorders such as Huntington's disease, Parkinson's disease, and various other movement as well as cerebellar disorders [3], [4]. SCA17 is a progressive and irreversible neurodegenerative disorder.

Genetically, in the case of SCA17 the CAG pattern (CAG trinucleotide repeat expansions) is repeated too many times, and disrupts the normal function of the encoding protein, a polyglutamine tract in the TATA-binding protein (TBP) gene [5] on chromosome 6q27. The normal repeat range is from 25 up to 42–45 units [6]. Pathogenic repeats from 46 to 63 are considered as expanded, whereas expansions from 43 to 48 CAG repeats are regarded as intermediate alleles with reduced penetrance [7] and CAG repeats from 49 to 66 as pathologic alleles with complete penetrance.

Neuropathological examination in *post mortem* brain tissues demonstrated cortical, subcortical, and cerebellar atrophy in SCA17 [2], [8]. At this, purkinje cell loss and gliosis, pseudohypertrophic degeneration of the inferior olive, marked neuronal loss and gliosis in the caudate nucleus, and in the medial thalamic nuclei were prominent features together with neuronal intranuclear inclusions stained with anti-TATA box-binding protein and antipolyglutamine antibodies [8].

On the neuroanatomical level, MRI studies revealed predominantly cerebellar atrophy among other brain structures in SCA17 patients [2], [7], [9], [10].

To investigate whether the expansion of CAG repeats of the TBP gene has an effect on the extent of brain atrophy in SCA17, we correlated genetic findings with cerebral volume measures.

## Materials and Methods

### Subjects

Sixteen SCA17 patients (mean age: 39.9±12.6 (SD) years, 10 male) were recruited from the outpatient movement disorders clinics at the Departments of Neurology of the Universities of Rostock and Luebeck in Germany; where the patients have been diagnosed and followed up on a regular basis. Total genomic DNA was extracted from peripheral blood leucocytes by standard protocols as described previously [5], [7]. The number of CAG repeats of the abnormal allele ranged from 44 to 55 triplets. The age at onset varied from 18 to 47 years and the duration of symptoms from 1 to 21 years (mean disease duration: 10.8±7.9 (SD) years). To measure the severity of ataxia, we used the International Cooperative Ataxia Rating Scale (ICARS), (mean ICARS score: 31.3±25.5(SD)). Regarding to Netravathi and colleagues, we defined patients' clinical disease progression as the quotient of the ICARS score divided by disease duration (ICARS/DD) at the time of examination [19].

All subjects underwent a screening test for dementia, the Mini-Mental State Examination Test (MMSE) [20]. Due to motor impairment in some subjects resulting in incomplete MMSE tests (e.g. drawing part), raw data were converted into per cent ranges. Details of demographic and clinical data are summarized in Table 1. Functional-morphological correlations between motor and neuropsychiatric signs have been reported before [9].

**Table 1:** Demographic and clinical data in SCA17 patients.

Number	Sex	Age (years)	DD (years)	Ataxia (ICARS)	CAG Repeats	MMSE(%)
1	M	34	20	59	52	96.7
2	M	42	7	9	51	100.0
3	W	28	7	51	55	89.3
4	M	46	19	50	54	50.0
5	M	42	3	0	49	96.7
6	M	40	5	4	51	100.0
7	W	66	21	41	46	60.7
8	W	22	1	36	44	100.0
9	W	45	20	59	54	75.0
10	W	54	7	54	51	60.7
11	W	50	18	71	45	13.3
12	M	51	20	44	54	33.3
13	M	23	4	3	54	100.0
14	M	34	16	18	49	100.0
15	M	20	2	0	54	100.0
16	M	42	2	1	49	96.7

**Abbr.:** DD, Disease Duration; ICARS, International Cooperative Ataxia Rating Scale; MMSE, Mini-Mental State Examination.

Sixteen age matched normal subjects (mean age: 40.75±10.8 (SD) years, 9 male) were not related to the SCA17 subjects. The following exclusion criteria were applied: a history of neurologic or psychiatric illnesses, prior exposure to neuroleptic agents or drug abuse, a medical history of hypertension, cardiovascular disease, or diabetes mellitus. The study was approved by the local ethics committee and written informed consent was obtained from all participating subjects in accordance with the Declaration of Helsinki [21] (http://www.wma.net/e/policy/b3.htm).

### MRI scanning and statistical analysis

All subjects underwent structural MRI imaging with a 1.5 T whole-body scanner (Symphony, Siemens, Erlangen, Germany) using a T1-weighted FLASH-3D MR sequence (echo time [TE] = 5 msec; repetition time [TR] = 15 msec; flip angle = 30°; isotropic voxel size = 1×1×1 mm^3^).

MR images for all subjects were analyzed on a commercially available Unix machine using a voxel-wise statistical approach. Images were processed and analysed with Statistical Parametric Mapping software (SPM5, Wellcome Department of Imaging Neuroscience, Institute of Neurology, UCL, London, www.fil.ion.ucl.ac.uk/spm) implemented in Matlab Version 7.6 (Mathworks, Sherborn, MA, USA) and the VBM5 toolbox (http://dbm.neuro.uni-jena.de/vbm). Applying a probabilistic framework, images were registered using linear (12-parameter affine) and non-linear transformations (warping), tissue classified, and bias corrected within the same generative model [22]. The following analyses were performed on gray matter segments that were multiplied by the non-linear components derived from the normalization matrix (modulated gray matter volumes). Finally, modulated gray matter images were smoothed with a Gaussian kernel of 12 mm full width at half maximum.

Using a general linear model, voxel-wise gray matter differences between the patient group and the respective control group were examined using independent-sample *t*-tests. To avoid possible edge-effects around the border between gray and white matter or cerebro-spinal fluid an absolute gray matter, threshold of 0.25 (absolute threshold masking) was used. For the statistical analysis, we employed in the first step an uncorrected threshold of *p*<0.001 across the whole brain. In the second step, to explore the association of regional brain volumes in SCA17 and length of the expanded CAG repeats, we performed a multiple regression analysis. The WFU PickAtlas [23] was used as an anatomical reference to assess the exact localisation of gray matter changes. Coordinates were reported in the standard anatomical space developed at the Montreal Neurological Institute (MNI).

Associations of clinical with genetic parameters or brain volumes were assessed by Pearson correlations. Post-hoc calculations and statistical analyses were performed on a Unix machine and SPSS software package (SPSS v17.0, Chicago, Illinois, USA). Given our hypotheses that the increase of the CAG repeat length is associated with a decrease in gray matter volume or rather an increase of the rate of disease progression, we used one-tailed Pearson' correlations for the post-hoc analysis. P-values of <0.05 were considered significant.

## Results

### Cerebral atrophy and its relation to the abnormal CAG repeat length in SCA17 patients

**Figure 1:**
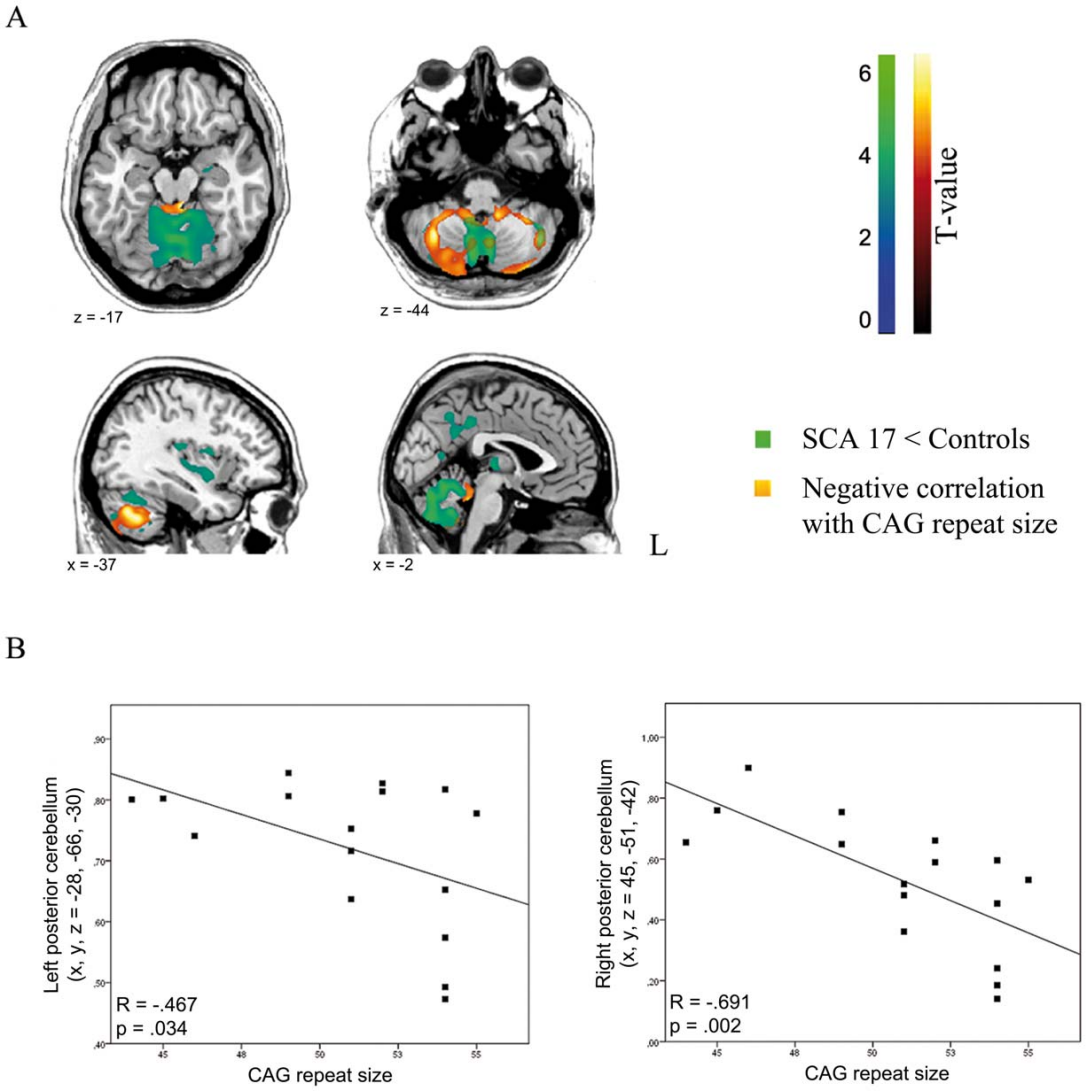
Grey matter changes and their correlation with CAG repeat size in SCA17 patients. A) Areas of grey matter volume reduction in SCA17 patients compared with age-matched healthy controls (green) and regression analysis with the abnormal CAG repeat length in SCA17 (orange). The color bar represents the T-values. B) Pearson correlations between expanded CAG size and brain volume measures revealing an inverse relationship with the left posterior (r = −.467, p = .034) and right posterior (r = .−691, p = .002) cerebellum.

Compared to the healthy control group, in SCA17 patients the largest cluster of gray matter volume reduction was found in the bilateral cerebellum posterior lobe, (Table 2, Figure 1). The regression analysis with the extended CAG repeats revealed a highly significant negative correlation with grey matter reduction in large parts of the cerebellum. The largest cluster of reduced gray matter volume (k_E_ = 12257) was detected in the right anterior lobe of the cerebellum extending to the other hemisphere, followed by the second largest one (k_E_ = 4982) in the cerebellar posterior lobe (Table 3, Figure 1).

**Table 2:** Categorical comparison of SCA17 patients and healthy controls.

Region	Left hemisphere					Right hemisphere				
	Coordinates			Z-score	k_E_	Coordinates			Z-score	k_E_
	x	y	z			x	y	z		
Cerebellum posterior lobe (VI)	−16	−80	−12	4.47	24685	11	−77	−14	3.26	24685
Cerebellum posterior lobe (CrII)	—	—	—	—	—	45	−51	−42	3.78	1074
Cerebellum anterior (R), posterior (L) lobe (VI)	−28	−66	−30	3.57	24685	32	−58	−29	3.67	836
Caudate	−12	20	−4	4.43	940	11	18	−1	4.19	773
Postcentral gyrus (BA 3,4)	−48	−27	41	3.78	439	48	−24	40	4.70	1322
Postcentral gyrus (BA 40)				—	—	53	−30	17	4.35	7582
Cingulate gyrus (BA 31)	−13	−41	40	4.08	3623	10	−45	39	3.61	3623

**Table 3:** Multiple regressions with abnormal CAG repeat length.

Region	Left hemisphere					Right hemisphere				
	Coordinates			Z-score	k_E_	Coordinates			Z-score	k_E_
	x	y	z			x	y	z		
Cerebellum anterior lobe (X)	−23	−37	−35	3.43	12257	20	−37	−33	4.30	12257
Cerebellum anterior lobe (III)	−5	−42	−28	4.12	65	4	−41	−26	3.95	764
Cerebellum posterior lobe (CrII)	−36	−58	−44	4.25	12257	44	−62	−45	3.92	4982

### Correlation of localized brain volume with clinical and genetic parameters

Post-hoc Pearson correlations between the length of the expanded CAG repeats and brain volumes showed a clear inverse correlation with grey matter atrophy values in left posterior (r = −.467, p = .034) and right posterior (r = .−691, p = .002) lobe of the cerebellum (Figure 1B).

Furthermore, we performed a correlation to determine the strength of the association between the expanded CAG repeat length and disease duration (DD)-adjusted ataxia scores measured by the ICARS (Ataxia/DD). By this, we found a significant inverse Pearson's correlation coefficient (r = −.492, p = .026) between the CAG repeat length and rate of disease progression. Correlations between other demographic or clinical data with the CAG repeat length did not reveal significant results.

## Discussion

Molecular neuroscience has clearly enhanced our pathobiological understanding of neurodegenerative disorders. Investigating a potential association of brain atrophy with genetic findings represents an innovative way to unravel genotype-phenotype relations in SCA17 patients. An observer-independent approach revealed a significant inverse correlation between the expanded size of CAG repeats and the extent of cerebral atrophy as well as the rate of disease progression in patients with SCA17. These findings might thus contribute to a better understanding of the etiologic mechanisms underlying SCA17.

SCA17, inherited in an autosomal dominant manner, is caused by an expanded CAG trinucleotide repeat in the TBP gene, coding for glutamine. TBP, a general transcription factor, is an essential component of the protein complex involved in RNA synthesis with ubiquitous expression, including the central nervous system [5]. Animal studies showed that complete loss of normal TBP function is not compatible with life. Pathophysiologically, it is thought that the neurodegenerative pathway is mediated by a toxic gain of function of the expanded polyglutamine stretch [11]. Regarding other SCA subtypes such as SCA1, 2, 3, 6 and 7, it has been reported that the CAG repeat size has not only a major effect on the age of onset but also on the phenotypic expression and its rate of progression [12]. Functional clinical measures correlated with the CAG repeat length, more in SCA3 than in SCA1 [13]. The frequently found correlation between length of CAG tracts and age of onset in polyglutamine disorders, could be also affirmed for SCA17 [2], [7].

On the neuroimaging level, there have been two earlier MRI reports in SCA, one found a correlation of CAG repeat length with one-dimensional or two-dimensional MRI measures of the pons and cerebellar vermis in SCA3 patients [14], whereas the other one failed to find a correlation between CAG repeat length and normalized brain volume in SCA1, 2 and 3 [15]. However, a large multicenter MRI study in SCA1, 3 and 6 revealed that abnormal length of CAG repeats was associated with a decreased brainstem, midbrain and putamen/caudate volume in SCA3, but only mild with pons volume in SCA1 [13].

To our knowledge, this is the first study linking cerebral atrophy and expanded CAG trinucleotid repeats in SCA17. MRI characteristics in SCA17 include atrophy of the cerebellum, cerebral cortex, brain stem and basal ganglia [8], [9], [16], [17], [18]. These studies also underlined the primary degeneration of cerebellar structures. Clinical motor and neuropsychiatric correlations demonstrated further the impact of the cerebellar degeneration on the cerebro-cerebellar network [9]. Our findings suggest that the rate of development of atrophy in the cerebellum, which is most affected in SCA17, depends on the size of the CAG repeats. Numerous CAG repeats seem to induce faster cerebellar atrophy and are therefore involved in the pathobiological progress of neurodegeneration in SCA17. Although the exact pathophysiological underlying cellular mechanisms of the expanded CAG repeats remains unknown and have to be elucidated in further studies, our results emphasize the essential role of the cerebellum and support the hypothesis of a distinct genotype-phenotype relationship in the pathobiology of SCA17.
