# Correction: CAG Repeats Determine Brain Atrophy in Spinocerebellar Ataxia 17: A VBM Study

**DOI:** 10.1371/annotation/e30b739b-114e-445a-b07b-7e2a8efa2668

**Published:** 2011-01-27

**Authors:** Kathrin Reetz, Alexandra Kleiman, Christine Klein, Rebekka Lencer, Christine Zuehlke, Kathrin Brockmann, Arndt Rolfs, Ferdinand Binkofski

The second author's name was originally misspelled. The correct spelling is: Alexandra Kleiman. The correct citation is: Reetz K, Kleiman A, Klein C, Lencer R, Zuehlke C, et al. (2011) CAG Repeats Determine Brain Atrophy in Spinocerebellar Ataxia 17: A VBM Study. PLoS ONE 6(1): e15125. doi:10.1371/journal.pone.0015125

